# COVID-19 and rheumatic autoimmune systemic diseases: report of a large Italian patients series

**DOI:** 10.1007/s10067-020-05334-7

**Published:** 2020-08-27

**Authors:** Clodoveo Ferri, Dilia Giuggioli, Vincenzo Raimondo, Massimo L’Andolina, Antonio Tavoni, Riccardo Cecchetti, Serena Guiducci, Francesco Ursini, Maurizio Caminiti, Giuseppe Varcasia, Pietro Gigliotti, Roberta Pellegrini, Domenico Olivo, Michele Colaci, Giuseppe Murdaca, Raffaele Brittelli, Giuseppa Pagano Mariano, Amelia Spinella, Silvia Bellando-Randone, Vincenzo Aiello, Silvia Bilia, Daiana Giannini, Tommaso Ferrari, Rodolfo Caminiti, Veronica Brusi, Riccardo Meliconi, Poupak Fallahi, Alessandro Antonelli, for the COVID-19 & ASD Italian Study Group

**Affiliations:** 1 Rheumatology Unit, School of Medicine, University of Modena & RE, Modena, Italy; 2 Rheumatology Clinic ‘Madonna dello Scoglio’ Cotronei, Crotone, Italy; 3 Rheumatology Outpatient Clinic, ASP-Vibo Valentia–Tropea Hospital, Tropea, Italy; 4 Clinical Immunology Unit, University of Pisa, Pisa, Italy; 5 Ospedale di Portoferraio, Livorno, Italy; 6 Rheumatology Unit, University of Florence, Florence, Italy; 7 Rizzoli Orthopaedic Institute Bologna, University of Bologna, Bologna, Italy; 8 UOD Reumatologia, Grande Ospedale Metropolitano, Reggio Calabria, Italy; 9 U.O.S. Reumatologia, Ospedale Castrovillari, Cosenza, Italy; 10 U.O.T. Specialistica Ambulatoriale ASP 201, Cosenza, Italy; 11 U.O.C. Medicina Interna “M.Valentini” P.O. Annunziata, Cosenza, Italy; 12 Rheumatology Outpatient Clinic, San Giovanni di Dio Hospital, Crotone, Italy; 13 Rheumatology Unit, University of Catania, Catania, Italy; 14 Department of Internal Medicine, San Martino Policlinic Hospital, University of Genoa, Genoa, Italy; 15 Department of Translational Research and New Technologies in Medicine and Surgery, School of Medicine, University of Pisa, Pisa, Italy; 16 Department of Clinical and Experimental Medicine, Immuno-Endocrine Section of Internal Medicine, Laboratory of Primary Human Cells, School of Medicine, University of Pisa, Via Savi, 10, I-56126 Pisa, Italy

**Keywords:** Arthritis, Autoimmune systemic diseases, Connective tissue diseases, COVID-19, Rheumatic diseases, SARS-CoV-2

## Abstract

**Introduction:**

Covid-19 infection poses a serious challenge for immune-compromised patients with inflammatory autoimmune systemic diseases. We investigated the clinical-epidemiological findings of 1641 autoimmune systemic disease Italian patients during the Covid-19 pandemic.

**Method:**

This observational multicenter study included 1641 unselected patients with autoimmune systemic diseases from three Italian geographical areas with different prevalence of Covid-19 [high in north (Emilia Romagna), medium in central (Tuscany), and low in south (Calabria)] by means of telephone 6-week survey. Covid-19 was classified as (1) *definite* diagnosis of Covid-19 disease: presence of symptomatic Covid-19 infection, confirmed by positive oral/nasopharyngeal swabs; (2) *highly suspected* Covid-19 disease: presence of highly suggestive symptoms, in absence of a swab test.

**Results:**

A significantly higher prevalence of patients with *definite* diagnosis of Covid-19 disease*,* or with *highly suspected* Covid-19 disease, or both the conditions together, was observed in the whole autoimmune systemic disease series, compared to “Italian general population” (*p* = .030, *p* = .001, *p* = .000, respectively); and for *definite + highly suspected* diagnosis of Covid-19 disease, in patients with autoimmune systemic diseases of the three regions (*p* = .000, for all comparisons with the respective regional general population)*.* Moreover, significantly higher prevalence of *definite + highly suspected* diagnosis of Covid-19 disease was found either in patients with various “connective tissue diseases” compared to “inflammatory arthritis group” (*p* < .000), or in patients without ongoing conventional synthetic disease-modifying anti-rheumatic drugs treatments (*p* = .011).

**Conclusions:**

The finding of a higher prevalence of Covid-19 in patients with autoimmune systemic diseases is particularly important, suggesting the need to develop valuable prevention/management strategies, and stimulates in-depth investigations to verify the possible interactions between Covid-19 infection and impaired immune-system of autoimmune systemic diseases.

**Table:** 

**Key Points** *• Significantly higher prevalence of Covid-19 is observed in a large series of patients with autoimmune systemic diseases compared to the Italian general population, mainly due to patients’ increased susceptibility to infections and favored by the high exposure to the virus at medical facilities before the restriction measures on individual movement.* *• The actual prevalence of Covid-19 in autoimmune systemic diseases may be underestimated, possibly due to the wide clinical overlapping between the two conditions, the generally mild Covid-19 disease manifestations, and the limited availability of virological testing.* *• Patients with “connective tissue diseases” show a significantly higher prevalence of Covid-19, possibly due to deeper immune-system impairment, with respect to “inflammatory arthritis group”.* *• Covid-19 is more frequent in the subgroup of autoimmune systemic diseases patients without ongoing conventional synthetic disease-modifying anti-rheumatic drugs, mainly hydroxyl-chloroquine and methotrexate, which might play some protective role against the most harmful manifestations of Covid-19.*

## Introduction

The recent outbreak due to a newly identified β-coronavirus (Covid-19, or SARS-CoV-2) spreads worldwide (today, May 21, 2020); > 5,000,000 cases are reported, and the number is increasing every day. About 30–70% of infected patients are asymptomatic (*asymptom-covid-19*); however, 20–50% need hospitalization [1, 2].

Within the subset of Covid-19 symptomatic patients (*sympt-Covid-19*) admitted to hospital, the most common symptoms at onset of illness were fever, cough, dyspnea, myalgia, or fatigue. Notably, 20–30% of *sympt-Covid-19* had upper respiratory tract symptoms such as coryza or other symptoms such as nausea, vomiting, diarrhea, dysgeusia, and anosmia. About 20–30% of hospitalized patients required intensive care unit admission for respiratory support; among them, 70–80% of patients were male, older than 65 years, and 30–50% had pre-existing comorbidities, such as hypertension (15–25%), diabetes (20–25%), obesity, and cardiovascular diseases (10–15%), or chronic obstructive pulmonary disease (10–15%) [2–6]. On the whole, increased risk to worse Covid-19 disease outcomes may be observed in immune-compromised patients [6–9]. Therefore, Covid-19 pandemic infection poses a serious challenge for the management of patients with “inflammatory autoimmune systemic diseases” (ASD). In fact, ASD patients represent a vast population of tens of millions of patients worldwide with compromised immune system and increased susceptibility to different types of viral and bacterial infections, frequently aggravated by ongoing immune modifier treatments [6–9].

On the other side, several anecdotal observations and cohort studies of ASD patients suggested the potential therapeutic role of some anti-rheumatic immune-modulating drugs (such as hydroxychloroquine and tocilizumab), which may potentially interfere with viral infection or the cytokine storm syndrome observed in Covid-19 in association with severe acute respiratory distress syndrome. Therefore, the immune-compromised condition along with the frequent administration of immune-suppressive treatments in ASD patients might prevent them from Covid-19 major complications [8, 10–13].

These apparently conflicting assumptions may be clarified by epidemiological studies on the actual incidence of Covid-19 in ASD patients, as well as by the results of ongoing trials on various anti-inflammatory/immune-modulating therapies in patients with Covid-19 [8, 10–15].

At the beginning of March 2020, the rapid diffusion of pandemic Covid-19 has induced the Italian government to take stringent measures aimed at slowing the spread of the virus. The restrictions on individual movement have compromised the regular face-to-face activities of outpatient clinics, mainly required for the tight monitoring of ASD patients undergoing immune-modulating treatments; the same restrictions also prevented the timely detection of symptoms suggestive of Covid-19 disease. In this context, we organized a multicenter telephone survey study aiming to identify subjects with overt/suspected Covid-19 disease in a large ASD patient population.

Here, we report the epidemiological and clinical findings, observed in our ASD patients during the 6-week survey period coincident with the most critical phase of pandemic Covid-19 in Italy.

## Patients and methods

The present observational multicenter cohort study aimed to investigate the prevalence of Covid-19 infection in a large series of ASD Italian patients, resident in three geographical areas of Italy, with different prevalence of Covid-19 infection (high prevalence in north, medium in central, and low in south, Italy), by means of a telephone 6-week survey (from March 15th, to April 25th) in order to evaluate the cumulative prevalence from January 2020 of overt/suspected Covid-19 disease in ASD.

Table 1 summarizes the main characteristics of ASD patients followed at the 13 tertiary referral centers of three regions of northern (Emilia Romagna), central (Tuscany), and southern (Calabria) Italy. A total of 1641 unselected ASD patients (F 1256, M 385, mean age 59.7 ± 13.2 years, mean disease duration 11.5 ± 8.3 years) were consecutively enrolled. The classification and clinical assessment of various ASD diseases were carried out by means of current international criteria [16]. Clinical-epidemiological and laboratory features, including comorbidities (obesity, hypertension, diabetes, renal, pulmonary, and cardiovascular involvement), were obtained from individual records of ASD patients. In addition, the following information were carefully collected by trained physicians during the telephone interview according to standardized symptom-assessment questionnaire:

1. updating of ASD clinical features, disease activity, and ongoing treatments of underlying comorbidities;
2. presence and duration of any signs/symptoms [fever (temperature > 99 °F), cough, dyspnea, myalgia, arthralgia, fatigue, coryza, nausea, vomiting, headache, diarrhea, dysgeusia, anosmia] of Covid-19 disease that had appeared within the last 2 months;
3. sudden worsening of pre-existing manifestations such as arthralgia, myalgia, fatigue, fever, skin lesions, and respiratory symptoms (i.e., cough and dyspnea) possibly due to ASD-related interstitial lung involvement;
4. results (if any) of oral/nasopharyngeal swabs for Covid-19 infection at polymerase-chain-reaction testing.

**Table 1:** Clinico-epidemiological features and treatments of 1641 patients recruited for the survey on COVID-19 and ASD

						Ongoing treatments						
Autoimmune systemic diseases	Pts no.	F/M	Age years mean ± SD	Dis Dur years mean ± SD	Known exposure to Covid-19	Only symptomatic^a^ %	At least one major drugs^d^ %	Steroids^b^ %	csDMARD %	bDMARD %	tsDMARD %	Others^c^ %
Total	1641	1256/385	60 ± 13	11 ± 8	45/1641	5	95	57	62	53	4	30
Rheumatoid arthritis	695	518/177	63 ± 13	12 ± 8	5/695	4	96	86	74	53	8	5
Psoriatic arthritis	208	124/84	56 ± 11	11 ± 8	6/208	3	97	19	69	93	2	3
Ankylosing spondylitis	35	10/25	50 ± 13	11 ± 9	0/35	3	97	20	37	69	-	9
Systemic sclerosis	438	384/54	60 ± 13	10 ± 7	18/438	1	99	39	34	12	-	99
Systemic lupus eryth.	76	71/5	48 ± 14	15 ± 10	6/76	18	82	74	71	13	3	22
UCTD	64	60/4	55 ± 12	11 ± 8	4/64	9	92	50	80	5	-	5
PM/DM	19	14/5	61 ± 13	8 ± 10	3/19	10	89	68	47	21	-	79
Sjögren syndrome	18	18/0	69 ± 14	12 ± 9	1/18	17	83	78	78	6	-	11
Miscellany^e^	88	57/31	56 ± 14	13 ± 9	2/88	16	84	84	74	75	3	48

*ASD* autoimmune systemic diseases, *csDMARD* conventional synthetic disease-modifying anti-rheumatic drugs (hydroxychloroquine, chloroquine, methotrexate, leflunomide, sulfasalazine, cyclosporine), *bDMARD* biological disease-modifying antirheumatic drugs (infliximab, adalimumab, etanercept, abatacept, tocilizumab, rituximab, anakinra, belimumab, canakinumab, certolizumab, golimumab, ixekizumab, sarilumab, secukinumab, ustekinumab, denosumab, apremilast), *tsDMARD* targeted synthetic disease-modifying anti-rheumatic drugs (tofacitinib, baricitinib), *UCTD* undifferentiated connective tissue diseases, *PM/DM* polymyositis/dermatomyositis

^a^Low dose (≤ 5 mg/day prednisone equivalent), non-steroidal anti-inflammatory drugs, and/or analgesics

^b^> 5 mg/day prednisone equivalent

^c^Other drugs: azathioprine, mycophenolate mofetil, cyclosporine, cyclophosphamide, IVIGs; vasoactive drugs (particularly for systemic sclerosis pts): iloprost, bosentan, macitentan, pilocarpine, selexipag, sildenafil, tadalafil, PGE, low dose aspirin

^d^High-dose steroids, csDMARD, bDMARD, tsDMARD, and/or others

^e^Mixed connective tissue disease, Behçet’s disease, idiopathic juvenile arthritis, enteropathic arthritis, sarcoidosis, polymyalgia rheumatica, systemic vasculitis, undifferentiated inflammatory arthritis

Subjects experiencing any symptom variations were invited to contact directly the interviewing physicians, after the call.

On these bases, Covid-19 was classified as

1. *definite* diagnosis of Covid-19 disease (*def-sympt-Covid-19)*: presence of symptomatic Covid-19 infection always confirmed by positive oral/nasopharyngeal swabs at polymerase chain reaction testing;
2. *highly suspected* Covid-19 disease (*suspect-sympt-Covid-19*): presence of fever (temperature > 99 °F) and/or known contact with Covid-19-infected individual, plus four or more symptoms, such as dry cough, sore throat, shortness of breath, dyspnea, sudden worsening of preexisting respiratory symptoms, anosmia, dysgeusia, nausea, vomiting, headache, diarrhea. This group comprises a significant number of patients characterized by manifestations highly suggestive of Covid-19 disease not confirmed by Covid-19 oral/nasopharyngeal swabs at polymerase chain reaction testing (because they were not submitted to the test, often due to limited availability of virological tests).

The results were analyzed performing the odds ratio (OR) by Java-Stat 2-way Contingency Table Analysis. STATA, and StatView were also used to evaluate other variables (data are expressed as mean ± 2SD).

Shapiro-Wilk test was used to evaluate the distribution of age and disease duration.

## Results

Main demographic and clinical features of the 1641 ASD Italian patients examined in this survey study are reported in Table 1. The ASD series encompasses 938 patients with “inflammatory arthritis” (rheumatoid arthritis, psoriatic arthritis, ankylosing spondylitis), 615 patients with “connective tissue diseases” [systemic sclerosis (SSc), systemic lupus, undifferentiated connective tissue disease, polymyositis/dermatomyositis, Sjogren’s syndrome], and miscellany of 88 subjects with less frequent ASD. The female/male ratio (3.26:1) of the whole series confirmed the well-known prevalence of female gender commonly observed in ASD, especially in connective tissue diseases.

With respect to ongoing treatments, the large majority of patients (95%) were taking at least one of the major anti-rheumatic immune-modifier drugs, namely, conventional synthetic disease-modifying anti-rheumatic drugs (csDMARD), biological disease-modifying anti-rheumatic drugs (bDMARD), and targeted synthetic disease-modifying anti-rheumatic drugs (tsDMARD). Patients with “connective tissue diseases” were more often treated with mycophenolate mofetil, methotrexate, some bDMARD (rituximab and belimumab), and/or vasoactive drugs [the latter more frequently employed in SSc patients] (Table 1), than patients with “inflammatory arthritis”.

Following the proposed classification criteria, *def-sympt-Covid-19* was recorded in 11 (0.7%) and *suspect-sympt-Covid-19* in 14 (0.8%) ASD patients; on the whole 25 patients with *def-sympt-Covid-19*, or *suspect-sympt-Covid-19*, were present among ASD patients (1.5%). This prevalence was higher than that observed in the Italian population of Covid-19-infected individuals [349/100,000 = 0.3%; data from the Italian Superior Institute of Health (ISS)] [17].

A lower mean age and a higher prevalence of female gender were observed in the ASD population with respect to the Italian Covid-19-infected population (60 vs 62 years; 76% vs 53%, respectively) [17].Clinically, mild-moderate Covid-19 syndrome was observed in 23 ASD patients without differences between *def-sympt-Covid-19* or *suspect-sympt-Covid-19*. Two patients developed severe Covid-19 disease, namely: (1) a 74-year-old female with inflammatory arthritis associated to Schnitzler’s syndrome developed *def-sympt-Covid-19* needing hospitalization for severe pneumonia, who recovered within a 3-week period; (2) a 65-year-old female with SSc (and lung fibrosis) with *def-sympt-Covid-19*, who died for severe acute respiratory distress syndrome, pulmonary venous/arterial thrombotic disease, and embolic *stroke.*

On the whole, significant higher prevalence of *def-sympt-Covid-19* was observed in patients resident in the two regions of north-central Italy (Emilia Romagna and Tuscany), compared to southern Italy (Calabria) (*p* = .000).

Table 2 shows the prevalence of *def-sympt-Covid-19* or *suspect-sympt-Covid-19*, observed in the ASD series compared to the prevalence of Covid-19 infection in the Italian, or regional, general population [17]. A significantly (*p* = .030) higher prevalence of *def-sympt-Covid-19* was observed in the whole ASD series, compared to the Italian general population; similarly, a significantly higher prevalence was observed for either *suspect-sympt-Covid-19* (*p* < .001) or when adding *def-sympt-Covid-19* with *suspect-sympt-Covid-19* (*p* = .001, and *p* = .000, respectively).

**Table 2:** Prevalence of COVID-19 in Italian patients with ASD compared to general and regional population

	Definite		*p*	OR value	Highly suspected	*p*	OR value	Definite + highly suspected	*p*	OR value
	Pts n.	*n. 11*	Mantel-Haenszel	95% CI	*n. 14*	Mantel-Haenszel	95% CI	*n. 25*	Mantel-Haenszel	95% CI
*ASD* vs Italian general population	1641	11	0.030	1.93 1.05–3.52	14	0.001	2.46 1.44–4.20	25	0.000	4.42 2.93–6.65
	100,000	349								
ASD vs population of 3 Italian regions										
ASD from Emilia Romagna	278.0	2.0	0.701	1.31 0.33–5.29	9	0.000	6.03 3.09–11.77	11	0.000	7.42 4.04–13.64
Emilia Romagna population	100,000	552								
ASD from Tuscany	*429.0*	6.0	0.000	6.35 2.80–14.36	3	0.681	1.27 0.41–3.96	9	0.000	3.86 1.98–7.51
Tuscany population	100,000	223								
ASD from Calabria	*934.0*	3.0	0.001	5.86 1.83–18.75	2	0.042	3.90 0.95–16.01	5	0.000	9.78 3.91–24.49
Calabria population	100,000	55								

*ASD* autoimmune systemic diseases

The prevalence of Covid-19 recorded in ASD patients resident in the three Italian regions considered in our survey was also matched with that reported in the corresponding regional general populations [17]. A significantly higher prevalence was observed for *def-sympt-Covid-19* in ASD patient subgroups resident in Tuscany (*p* = .000) and Calabria (*p* = .001) (with respect to the regional population), but not for Emilia Romagna (this last result likely due to the lower number of recruited patients in this region, and the frequent unavailability of swab test). A significantly higher prevalence was found for *suspect-sympt-Covid-19* in ASD from Emilia Romagna (*p* = .000) or Calabria (*p* = .042). However, the prevalence of the *def-sympt-Covid-19 + suspect-sympt-Covid-19* patients was significantly increased in each of the above mentioned regions in ASD patients (*p* < .000, for each comparison).

The comparison of Covid-19 infection, among the subgroups of ASD patients according to diagnosis (“connective tissue diseases” vs “inflammatory arthritis”), or absence/presence of major comorbidities, or different treatment categories, revealed some significant differences (Table 3):

- the group of patients with “connective tissue diseases” showed higher prevalence of either *suspect-sympt-Covid-19* (*p* < .000) or *def-sympt-Covid-19 + suspect-sympt-Covid-19* (*p* < .000), compared to the complex of “inflammatory arthritis”;
- the prevalence *of def-sympt-Covid-19* was higher in ASD without major comorbidities (*p* = .015);
- patients without csDMARD revealed significantly higher prevalence of either *suspect-sympt-Covid-19* (*p* = .003) or *def-sympt-Covid-19 + suspect-sympt-Covid-1*9 (*p* = .011).

**Table 3:** Comparison of Covid-19 prevalences among different subgroups of ASD patients

	Definite		*p*	OR value	Highly suspected	*p*	OR value	Definite + highly suspected	*p*	OR value
	Pts n.	*n. 11*	Mantel-Haenszel	95% CI	*n. 14*	Mantel-Haenszel	95% CI	*n. 25*	Mantel-Haenszel	95% CI
Connective tissue dis + others	703	7	0.162	2.35 0.68–8.05	13	0.000	17.65 2.30–135.27	20	0.000	5.46 2.04–14.63
vs RA, PsA, AS	938	4			1			5		
comorbidities -	288	5	0.015	3.97 1.20–13.09	3	0.702	1.28 0.36–4.63	8	0.056	2.24 0.96–5.25
vs ASD comorbidities +	1353	6			11			17		
csDMARD -	647	5	0.218	1.28 0.39–4.22	11	0.003	5.71 1.59–20.56	16	0.011	2.77 1.22–6.32
vs ASD cDMARD +	994	6			3			9		
bDMARD & tsDMARD −	793	5	0.848	0.89 0.27–2.93	10	0.082	2.7 0.84–8.63	15	0.239	1.62 0.72–3.62
vs ASD bDMARD & tsDMARD+	848	6			4			10		
ASD & others drugs +	551	4	0.844	1.13 0.33–3.88	8	0.061	2.66 0.92–7.71	12	0.124	1.84 0.84–4.07
vs ASD & others drugs −	1090	7			6			13		

*ASD* autoimmune systemic diseases, *RA* rheumatoid arthritis, *PsA* psoriatic arthritis, *AS* ankylosing spondylitis, *csDMARD* conventional synthetic disease-modifying anti-rheumatic drugs (methotrexate, leflunomide, sulfasalazine, hydroxychloroquine, cyclosporine, chloroquine), *bDMARD* biological disease-modifying antirheumatic drugs (infliximab, adalimumab, etanercept, abatacept, tocilizumab, rituximab, anakinra, belimumab, canakinumab, certolizumab, golimumab, ixekizumab, sarilumab, secukinumab, ustekinumab, denosumab), *tsDMARD* targeted synthetic disease-modifying anti-rheumatic drugs (tofacitinib, baricitinib); other drugs: azathioprine, mycophenolate mofetil, cyclosporine, cyclophosphamide, IVIGs, and/or vasoactive drugs (particularly for systemic sclerosis pts): iloprost, bosentan, macitentan, pilocarpine, selexipag, sildenafil, tadalafil, PGE, low-dose aspirin

## Discussion

The present survey study investigated the prevalence of Covid-19 in a large ASD patient series from three distinct regions of Italy characterized by different pandemic spread. A significantly higher prevalence of *def-sympt-Covid-19* was observed both in the whole ASD population (with respect to the Italian general population) and in the regional ASD subgroups of Tuscany, or Calabria, patients (when compared with the corresponding regional prevalence of Covid-19). The relatively low prevalence of *def-sympt-Covid-19* in the Emilia Romagna might be probably associated with the lower number of ASD patients evaluated in this region compared to those from Tuscany and Calabria, and the frequent unavailability of swab test. These significant differences are reinforced by the consideration that we have compared the prevalence of symptomatic Covid-19 ASD patients, with that of Covid-19-infected patients from the general population, that comprises also about 17–20% of not symptomatic individuals (as per ISS evaluation) [17]. In addition, the majority of subjects screened for Covid-19 were among those with high risk of infection such as health professionals. So, the differences might be more pronounced if a swab test screening would have been performed in ASD patients.

Significant differences were also found in both ASD patients with *suspect-sympt-Covid-19* and in the entire group of *def-sympt-Covid-19+suspect-sympt-Covid-19* ASD. Patients with *suspect-sympt-Covid-19* are characterized by a number of signs and symptoms highly suggestive of Covid-19; therefore, they should be clinically considered, at least provisionally, as true Covid-19 even in the absence of oral/nasopharyngeal swabs due to limited availability of virological tests at the time of the survey.

This assumption is also suggested by the observation of two patients previously classified as *suspect-sympt-Covid-19*, who subsequently revealed positive at swab testing during the survey period and were therefore classified as *def-sympt-Covid-19*.

Of note, the patients’ awareness of the risks inherent in their chronic illness along with the frequent physical limitations, all together, may lead to very cautious patients’ lifestyle, that in the case of the ongoing pandemic has certainly reduced the risk of contracting Covid-19 measures aimed at slowing the spread of the virus. However, the statistically increased prevalence of Covid-19 observed in our large series of patients with ASD certainly related to the increased susceptibility to infections has been favored by the high exposure to the virus during the frequent contacts with medical facilities and/or hospitalizations before the restriction measures on individual movement.

The higher prevalence of Covid-19 in ASD compared to that found in Italian general population was further emphasized by some demographic observations; in particular, ASD patients showed lower mean age, as well as a higher percentage of females. These findings are in counter tendency with respect to the epidemiology of Covid-19 symptomatic patients, which are prevalently male, aged > 60 years.

Accordingly to the different diffusion of Covid-19 in the Italian territory with a clear-cut north-south gradient, the ASD patients of north-central Italian macro-area (Emilia Romagna + Tuscany) showed higher prevalence of Covid-19 than those of southern Italy (Calabria).

Previous clinical investigations focusing on patients with various ASD complicated by Covid-19 reported in the world literature are summarized in Table 4 [18–33]; they include 4 single case observations [18–21], 3 cohort studies [22–25], a population-based study [32], and 6 survey studies [26–31, 33]. Overall, these previous reports indicated a prevalence of Covid-19 frequently comparable with those observed in the general population of corresponding geographical areas, particularly for patients series with chronic arthritis [26, 27], while increased percentage of Covid-19 affected patients with connective tissue diseases, namely systemic lupus [28] or large vessel vasculitis [29]. In all cases, the ongoing immune-modifier treatments, especially bDMARD, did not affect the outcomes of symptomatic, generally mild Covid-19 disease.

**Table 4:** Review of the literature on Covid-19 and autoimmune systemic diseases (ASD)

Authors	Ref. no.	Country	Type of study	Diseases	Female %	Pts n.	Covid-19			Pneumonia	Hospitalization	Death	Covid-19% prevalence^a^
							Total (%)	Definite	Highly susp.				
Mihai et al.	[18]	Switzerland	Case report	SSc^b^	1	1	1	1	0	0	1	0	-
Jones et al.	[19]	US	Case report	Kawasaki disease	1	1	1	1	0	0	1	0	-
Guilpain et al.	[20]	France	Case report	GPA^f^	1	1	1	1	0	1 (ARDS)	1	0	-
Beydon et al.	[21]	France	Case report	Myositis^d^	-	1	1	1	0	1	1	0	-
Mathian et al.	[22]	France	Cohort	SLE + HCQ	76	17	17	17	0	13 (ARDS 5)	14	2	-
Haberman et al.	[23]	US	Cohort	arthritis, IBD	57	86	86	59	27	7	14	1	-
Verdoni et al.	[24]	Italy	Cohort	Kawasaki-like	30	110	10	10	0	0	10	0	-
Cheng et al.	[25]	China	Cohort	RA, SSc	80	5	5	5	0	5	5	0	-
Monti et al.	[26]	Italy	Survey	RA, SpA, PsA	68	320	8 (2.5)	4	4	0	1	0	0.76 Lombardy
Favalli et al.	[27]	Italy	Survey	RA, pA, PsA, JIA	70	530	3 (0.6)	3	81, 15.2%^e^	0	0	0	0.76 Lombardy
Bozzalla- Cassione et al.	[28]	Italy	Survey	SLE	84	165	12 (7.2)	4	8	1 (ARDS 1)	1	0	0.76 Lombardy
Tomelleri et al.	[29]	Italy	Survey	GCA/TA	71.5	162	4 (2.5)	2/2	0	1/0	1/0	0	0.76 Lombardy
Emmi et al.	[30]	Italy	Survey	ASD	74	527	1 (0.2)	1^c^	(12)^c^	1	1	0	0.20; p: ns Tuscany
Conticini et al.	[31]	Italy	Survey	ASD	nd	859^a^	2 (0.2)	2	nd	2	3	0	nd
Quartuccio et al.	[32]	Italy	Population-based study	ASD^g^	66.9	1051	4 (0.4)	4	nd	2	2	0	0.20; p: ns province of Udine Italy
Zen et al.	[33]	Italy	Survey	ASD	78.6	916	148^h^ (16.1)	nd	nd	nd	2	0	nd
Present study	-	Italy	Survey	ASD	77	1641	25 (1.5)	11	14	2	1	1	0.39; *p* = 0.030 Italy

*ARDS* acute respiratory distress syndrome, *GCA* giant cell arteritis, *GPA* granulomatosis with polyangiitis, *HCQ* hydroxychloroquine, *IBD* inflammatory bowel diseases, *JIA* juvenile idiopathic arthritis; # prevalently chronic arthritis; *nd* not done, *PsA* psoriatic arthritis, *RA* rheumatoid arthritis, *SLE* systemic lupus erythematosus, *SpA* ankylosing spondylitis, *SSc* systemic sclerosis, *TA* Takayasu arteritis

^a^Covid-19 prevalence in the general population of corresponding geographical area (data from ref. no. 17)

^b^57-year-old women with lung involvement undergoing tocilizumab treatment

^c^12 pts. with symptoms ‘compatible’ with Covid-19 (1/7 swab tested positive)

^d^Secondary to Covid-19

^e^Referred to a subgroup of patients with mild symptoms of viral infection suggesting a possible underestimation of the real incidence of Covid-19 in rheumatic patients

^f^Granulomatosis with polyangiitis undergoing rituximab treatment

^g^Mainly chronic arthritis undergoing bDMARD or tsDMARD

^h^Symptomatic, any symptom

Of note, the statistical analysis revealed a higher prevalence of Covid-19 in the group of patients with “connective tissue diseases”, when compared with patients affected by different “inflammatory arthritis”, possibly due to more pronounced immune system dysfunction present in the first patients’ group; in agreement with the increased prevalence of Covid-19 observed in a series of patients with systemic lupus [28], or large vessel vasculitis [29].

Another interesting finding was the higher prevalence of Covid-19 in ASD without concomitant comorbidities; this unexpected observation may deserve further investigations, and however reinforce the concept that the immune-dysfunction is the main reason of the higher prevalence of Covid-19 infection in ASD patients.

Similarly, it is very difficult to explain the increased prevalence of Covid-19 in patient without ongoing csDMARD treatments; tentatively, we can hypothesize some protective role of long-term administration of any of these medications (perhaps, hydroxyl-chloroquine) towards Covid-19 infection, while presence/absence of other immune-modulating drugs, mainly bDMARD and tsDMARD, seems to be not relevant for the development/outcome of Covid-19, in agreement with data reported in previous studies (Table 4).

Overall, the results of the present survey including a wide spectrum of ASD, i.e., “connective tissue diseases”, and “inflammatory arthritis”, seem to confirm the relatively benign outcomes of Covid-19 in patients with ASD [23–31], considering that only one patient with SSc complicated by lung fibrosis died among 25 subjects with *def-sympt-Covid-19* or *suspect-sympt-Covid-19*. However, the increased prevalence of Covid-19 in ASD is in keeping with the well-known susceptibility of immune-compromised patients towards all infectious pathogens. Covid-19 is still scarcely known as regards the pathogenesis and clinical course of acute manifestations. Moreover, the few data available to now, as regards both pathological and immunological changes, are insufficient to predict the long-term effects of the infection, particularly for patients with profound immune system dysfunction such as ASD. In this respect, some manifestations of severe Covid-19 are comparable to that detectable in many ASD; in particular, the interstitial lung and diffuse vascular injury, that although evolving more quickly in severe Covid-19 disease, seems to reproduce the main pathological alterations of SSc [34, 35].

Our study may present some limitations, mainly with regards to the modality of patients’ data recording and the lack of proper virological testing in patients with highly suspected Covid-19. The limitations inherent to telemedicine, largely used during the pandemic restrictions on individual mobility, might be balanced at least in part by the good compliance of patients, interviewed by the same physicians who normally followed them in the face-to-face outpatient visits. While the classification of Covid-19 as highly suspected, on the basis of clear-cut clinical symptoms, should be confirmed by patients’ follow-up study with validated antibody tests able to detected previous exposure to Covid-19.

In conclusion, the finding of a higher prevalence of Covid-19 in immune-compromised ASD patients is particularly challenging for at least two aspects: (a) firstly, it suggests the need to develop valuable prevention and management strategies for ASD patients particularly vulnerable during the ongoing Covid-19 pandemic or its possible re-exacerbation; (b) it stimulates in-depth investigations to verify the potential interactions between Covid-19 infection and impaired immune system of ASD that may affect the natural course of both disorders.
